# LASP1, a Novel Protein in Spermatozoa and Acrosome Reaction

**DOI:** 10.1002/mrd.70127

**Published:** 2026-07-06

**Authors:** Alicia Bender, Kilian Andress, Michael R. Boesl, Claudia Staib, Adriane Woehl‐Wenigerkind, Burkhard Kneitz, Hendrik Bartolomaeus, Alma Zernecke, Elke Butt

**Affiliations:** ^1^ Institute for Experimental Biomedicine II University Hospital Würzburg Würzburg Germany; ^2^ Institute of Experimental Biomedicine I, University Hospital and Rudolf Virchow Center University of Würzburg Würzburg Germany; ^3^ Department of Obstetrics and Gynaecology University Medical Center Würzburg Würzburg Germany; ^4^ Department of Urology and Pediatric Urology University Hospital Würzburg Josef–Würzburg Germany

**Keywords:** CD44, F‐actin, LASP1, protein kinase G, spermatozoa

## Abstract

LIM and SH3 protein 1 (LASP1) is a widely expressed scaffold protein associated with actin‐rich structures. In *Lasp1*‐knockout mice, reproductive defects such as longer intervals between litters and reduced pup numbers were observed. Consistently, data from the Human Protein Atlas show strong LASP1 expression in spermatids, suggesting a reproductive role. Mammalian fertilization involves sequential processes of sperm activation, capacitation, and acrosome reaction, driven by bicarbonate‐mediated PKA activation, CNP‐regulated PKG stimulation, F‐actin polymerization, Ca^2+^‐signaling, and MAPK signaling pathways ‐ culminating in membrane fusion and CD44 cluster formation for interaction with the egg. In this context, we observed differential isoform expression of PKG in human and mice. Our data provide the first evidence of LASP1 presence in the head, midpiece, and tail of human and mouse spermatozoa. During capacitation, LASP1 undergoes dynamic changes, co‐localizes with F‐actin and stabilizes the equatorial ring segment. In mice, loss of LASP1 did not affect sperm morphology, motility or chemotaxis, but led to changes in protein phosphorylation, incomplete capacitation, and reduced hyaluronan receptor surface expression, revealing a novel role of LASP1 in sperm fertilization.

## Graphical abstract

LASP1 is expressed in spermatozoa, binds to F‐actin, and builds up the equatorial ring structure during acrosome reaction, which is important for hyaluronan receptor surface presentation.

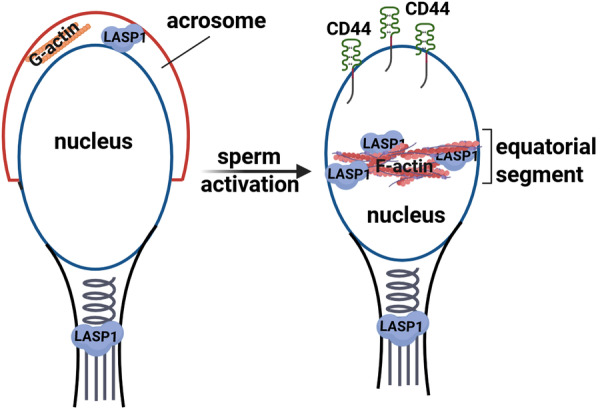

## 1 Introduction

LIM and SH3 protein 1 (LASP1) a ubiquitously expressed scaffolding protein, is predominantly associated with dynamic actin‐rich structures such as focal adhesions, and is involved in cell migration, adhesion, and proliferation (Butt et al. 2022; Ruggieri et al. 2017). LASP1 is overexpressed in various types of cancer and contributes to tumor progression and metastasis (Butt et al. 2022). New findings suggests that it is involvement in transcriptional regulation and nuclear signaling via stabilization AP1 (Endres et al. 2016), Snail1 (Liu et al. 2025) and Ago2 (Tilley et al. 2020) as well as through binding to AKT1 (Butt et al. 2020).

LASP1 possesses two known phosphorylation sites: Phosphorylation at serine 146 (S146) by protein kinase A and G (PKA and PKG) modulates the interaction of LASP1 with actin and other binding partners, thereby influencing cytoskeletal dynamics and cell motility (Butt et al. 2003). Phosphorylation at tyrosine residue Y171 by Src, Lyn and Abl is involved in cancer‐related signaling pathways and alters the localization and function of LASP1 (Butt et al. 2020; Lin et al. 2004).

In *Lasp1*‐knockout mice, we observed a prolonged time between litters and a reduced number of pups. Data from the Human Protein Atlas (https://www.proteinatlas.org) indicate a high LASP1 expression in spermatids.

Fertilization in mammals is a complex, highly regulated process that begins with sperm activation, followed by sperm capacitation and culminates in the acrosome reaction, enabling the sperm to penetrate the oocyte (Ikawa et al. 2010). In short, changes in bicarbonate levels are central to the sperm activation process. These stimulate PKA (Baro Graf et al. 2020) and thus initiate flagellar beating and motility. Capacitation is characterized by F‐actin polymerization and membrane remodeling (Gervasi et al. 2018), opening of CatSper channel and Ca^2+^ fluxes (Hwang and Chung 2023), stimulation and hyperactivation of PKG (Kaupp et al. 2008) and tyrosine phosphorylation of proteins by MAPKs (Kumar et al. 2024). During the exocytotic acrosome reaction, the outer acrosomal membrane fuses with the sperm plasma membrane. CD44 (hyaluronan receptor) forms clusters on the surface (clinically used in ICSI–HA binding assays) and enables interaction with hyaluronan in the cumulus matrix, thereby facilitating access of sperm to oocytes (Huszar et al. 2003; Merc et al. 2021).

Based on the regulatory impact of LASP1 on actin dynamics, we suspected a functional role of LASP1 in the tightly regulated sperm fertilization process. The results of our study provide, for the first time, data on the presence of LASP1 in spermatozoa and evidence for a functional role in the acrosome reaction and the surface expression of the hyaluronan receptor (CD44).

## 2 Results

### 2.1 LASP1 is Expressed in Human and Mouse Spermatozoa

**Figure 1:**
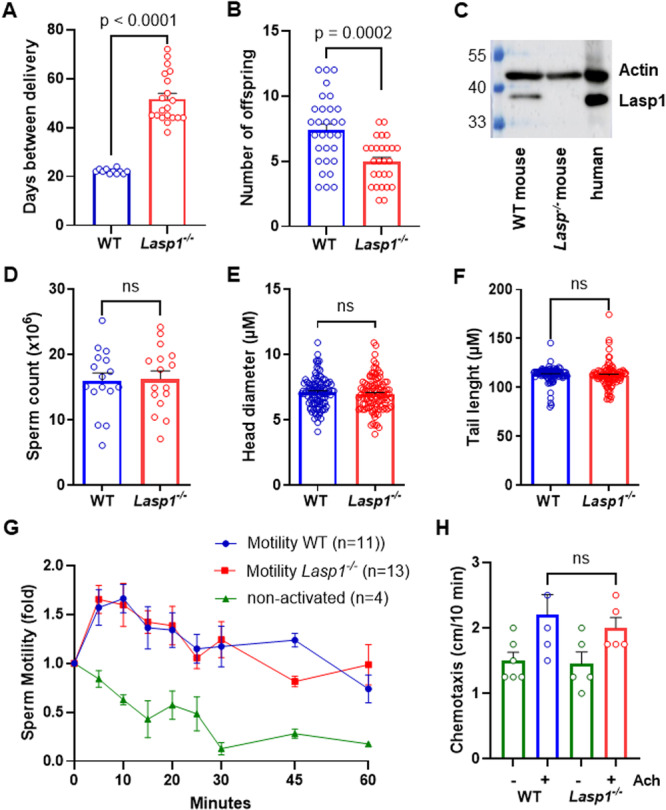
Morphometric analysis and reproductive function of spermatozoa from wild‐type (WT) and LASP1 knockout mice (*Lasp1* ^*‐/‐*^ *).* (A) The days between two litters (1–2 and 2–3) from 13 wild‐type (WT) and 18 *Lasp* ^*‐/‐*^ mated mice were counted. (B) Number of offspring from litter 2 and 3 of 15 WT and 14 *Lasp* ^*‐/‐*^ mice. (C) Western blot analysis of 2 Mio sperm per lane of WT, *Lasp* ^*‐/‐*^ and human sperm; β‐Actin served as loading control. (D) Sperm count of 16 WT and 16 *Lasp* ^*‐/‐*^ mice age 12–16 weeks. (E) Average head size and (F) tail length of 50 sperms from 10 WT and 10 *Lasp* ^*‐/‐*^ mice. (G) Time‐dependent analysis of WT and *Lasp1* ^*‐/‐*^ sperm motility after sperm activation with calcium/bicarbonate/albumin. (H) Linear swimming distances of WT and *Lasp1* ^*‐/‐*^ spermatozoa in the direction of an acetylcholine (Ach) gradient. A, B, D–F: Statistical differences were examined by unpaired Student's t‐test. Data are presented as mean ± SEM. G, H: Statistical differences were examined by Mann‐Whitney test. Data are presented as mean ± SEM.

A prolonged litter time in mice deficient in LASP1 was evidenced by a retrospective analysis of individual breeding cards: While WT control mice gave birth on average every 22 ± 0.3 day (*n* = 11), the time between two litters in LASP1 knockout mice strain increased to 54 ± 2.3 days (*n* = 18) (Figure 1A). In parallel, the total number of offsprings decreased from an average of 7.4 ± 0.5 pups (*n* = 30) to 4.9 ± 0.3 pups (*n* = 31) (Figure 1B).

Taken together, these data raise the question of whether LASP1 is expressed in spermatozoa and whether the protein directly affects sperm function and fertilization. Western blot analysis of human and mouse sperm verified expression of LASP1 in mammalian sperm (Figure 1C).

Of note, no Lasp1 expression was detected in mouse oocytes (Supporting Information Figure S1).

### 2.2 No Differences in Basal Sperm Parameters Between WT and LASP1‐KO Mice

Overall sperm production was unaltered between WT and LASP1 knockout‐mice (Figure 1D). Similarly, head area (Figure 1E) and tail length (Figure 1F) were unvaried. No visual sperm defects were observed.

Given that LASP1 regulates actin dynamics and cell motility (Pollitt et al. 2020), and that F‐actin is expressed in both the head and tail of spermatozoa (Breitbart et al. 2005), we hypothesized that Lasp1‐depletion affects sperm motility. However, computer‐assisted sperm analysis (CASA) revealed unaltered motility of *Lasp1* ^−/−^ spermatozoa under capacitation conditions (Figure 1G). Under non‐capacitation conditions, percentages of static (48%–53%), slow (0%), motile (37%–41%), and progressive (15%–16%) spermatozoa were unchanged between WT and *Lasp1* ^*‐/‐*^ mice. For chemotaxis analysis, swimming distance in response to and in the direction of an acetylcholine (ACh) gradient was measured. Chemotactic distance was not different, neither under non‐stimulated control conditions nor towards chemoattractant (Figure 1H).

### 2.3 LASP1 is Localized Within the Head, Midpiece, and Tail in Mammalian Spermatozoa and Interacts With Actin at the Equatorial Ring Structure After Capacitation

**Figure 2:**
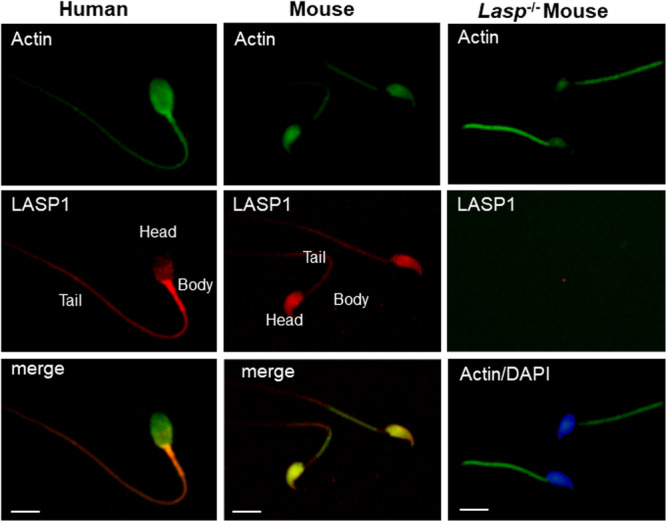
LASP1 expression in human and mouse spermatozoa. Sperm were fixed in 95% ice‐cold ethanol and stained for nucleus (DAPI), actin (Phalloidin‐green) and LASP1 (#pan1418 with Cy3‐red). Immunofluorescence displays LASP1 expression in head, midpiece (body) and tail of mammalian spermatozoa—co‐localized with actin (merge). LASP1 immunostaining is absent in Lasp‐/‐ mice. Scale bar: 10 µm.

Immunofluorescence staining of LASP1 in non‐activated human and mouse spermatozoa revealed co‐localization of the protein with actin in sperm head, midpiece, and tail (Figure 2). *Lasp* ^*‐/‐*^ mice show no LASP1 immunofluorescence confirming specificity of the antibody.

**Figure 3:**
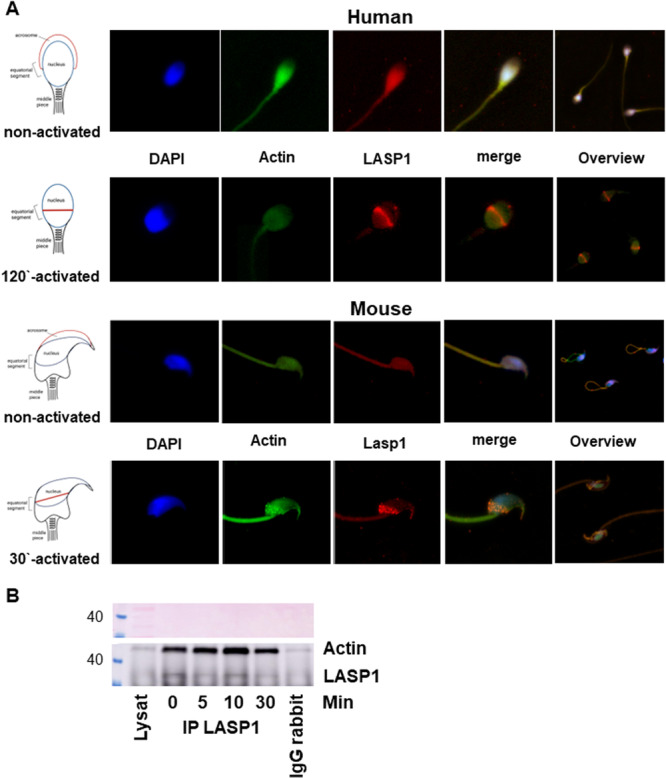
LASP1 localization before and after capacitation in mouse and human spermatozoa. Smear sperm preparations were fixed in 95% ice‐cold ethanol and stained for nuclei with DAPI (blue), actin (phalloidin‐green) and LASP1 (#pan1418 with Cy3‐red). After activation, human and mouse spermatozoa show co‐localization of LASP1 and actin at the equatorial segment (merge).

During mammalian sperm capacitation the outer acrosomal membrane vesiculates, and an equatorial ring segment is stabilized particularly by dynamic actin/cytoskeleton remodeling and G‐actin to F‐actin polymerization (Breitbart and Finkelstein 2018). In human and mouse spermatozoa, LASP1 is displaced from the acrosomal region and translocated to the developing equatorial ring where it associates with F‐actin (Figure 3A). Time‐dependent immunoprecipitation with LASP1 performed to investigate actin binding during mouse sperm and G/F polymerization revealed enhanced binding of LASP1 to polymerized F‐actin after 10 min of activation, that subsequently decreased after 30 min (Figure 3B). This is consistent with a rapid depolymerization of F‐actin back to G‐actin during acrosome reaction to enable membrane fusion and oocyte penetration.

### 2.4 LASP1‐Deficient Mouse Sperm Exert Reduced Acrosome Reaction, Hyaluronan Receptor Expression and Fertility

**Figure 4:**
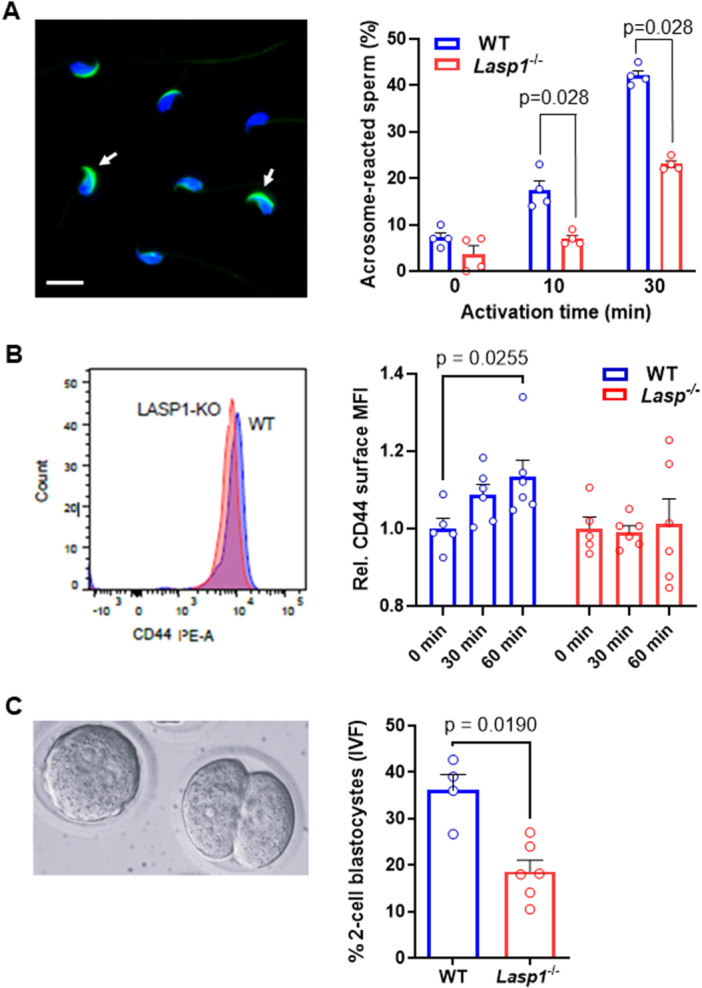
Quantification of the acrosome reaction in WT and LASP1‐deficient spermatozoa. (A) Left site: Exemplary mouse sperm acrosome staining by PNA‐488. Intact acrosome is stained in green (arrows). Nuclei are counterstained in blue (DAPI). Scale bar: 10 µM. Right site: Time‐dependent acrosome reaction in sperm of wild‐type (WT) and LASP1‐knockout (*Lasp* ^*‐/‐*^ *)* after stimulation with calcium/bicarbonate/albumin. A total of 120–150 spermatozoa was visually analyzed for no, partial or total loss of PNA staining. Only spermatozoa with complete loss of PNA‐staining were counted. Statistical differences were examined by Mann Whitney test. Data are presented as mean ± SEM (*n* = 4). (B) Flow cytometric analysis of hyaluronan receptor (CD44) expression on the surface of sperm from wild‐type (WT) and *Lasp1* ^*‐/‐*^ mice 30 and 60 min after activation. Statistical differences were examined by two‐way ANOVA test. Data are presented as mean ± SEM (*n* = 6). (C) In‐vitro‐fertilization of wild‐type oocytes with WT (*n* = 4) and LASP1‐deficient sperm (*n* = 6). For each experiment, 50–100 oocytes were incubated with 1.7–3.5 ×105 spermatozoa (ratio 1:3500), and two‐cell blastocysts were counted after 24 h. The experiments were conducted individually. Statistical differences were examined by Mann‐Whitney test. Data are presented as mean ± SEM.

Given the expression of LASP1 in mammalian sperm and its predominant localization in the equatorial ring segment, we next investigated whether loss of LASP1 affects capacitation. Peanut agglutinin (PNA) was used to detect the acrosome status in mouse spermatozoa. PNA is specific for d‐galactose beta 1‐‐‐‐3 N‐acetyl‐d‐galactosamine present only in the intact outer membrane (Lybaert et al. 2009). Quantification of PNA‐negative sperm heads after calcium/albumin treatment revealed significant less (≈50%) acrosome reaction in LASP1 deficient mice (Figure 4A).

After completing plasma membrane remodeling, the transmembrane hyaluronan receptor CD44 is accessible on the inner acrosome membrane of the mature sperm and allows binding to the hyaluronan‐rich cumulus oophorus surrounding the egg–a critical step in fertilization (Huszar et al. 2003). Flow cytometric analysis of CD44 surface expression on capacitated sperm from WT and *Lasp1* ^‐/‐^ mice revealed a significant increase in hyaluronan receptor expression upon activation only in WT‐spermatozoa (Figure 4B).

To test whether this reduced CD44 expression has any impact on fertility, we performed in‐vitro‐fertilization (IVF) experiments with WT‐oocytes and WT or *Lasp1* ^*‐/‐*^ sperm. Fertility was scored at the two‐cell stage embryos after overnight culture. The average fertility rate was significantly lower with *Lasp1* ^‐/‐^ sperm (37.5 ± 5.9% vs. 18.1 ± 5.5%, Figure 4C). This rate is much lower than the live‐birth rate in WT female mice paired with *Lasp1* ^*‐/‐*^ male, however, in mice IVF and embryo culture typically produce fewer high‐quality blastocysts (and lower live‐birth rates) than natural in vivo fertilization because the laboratory environment reproduces only part of the physiological reproductive tract environment (Gualtieri et al. 2024). Therefore, IVF fertilization rate reflects average sperm competence whereas in vivo, cumulus cells and the oviduct environment can compensate for sperm defects.

### 2.5 Human and Mouse Sperm Differ in PKG Isoform Expression

Protein phosphorylation, particularly tyrosine phosphorylation, is a key regulatory mechanism during sperm capacitation, with various protein kinases playing crucial roles. PKA catalytic subunit exists in a unique sperm‐specific isoform named Cα2, a splice variant that cannot be myristoylated (Nolan et al. 2004). PKG signaling is linked to the regulation of ion channels (e.g., CatSper and K^+^ channels) and modulates flagellar beat patterns (Kaupp et al. 2008).

Here, we show for the first time that human and mouse sperm differ with regard to their PKG isoforms and their localization: whereas murine sperm contain PKGIβ, human sperm express the more cGMP‐sensitive isoform PKGIα (Supporting Information Figure S2)—likely exhibiting more sensitive, redox‐responsive, and dynamically regulated cGMP signaling.

### 2.6 LASP1‐Knockout Sperm Exhibit Reduced Serine and Tyrosine Phosphorylation

**Figure 5:**
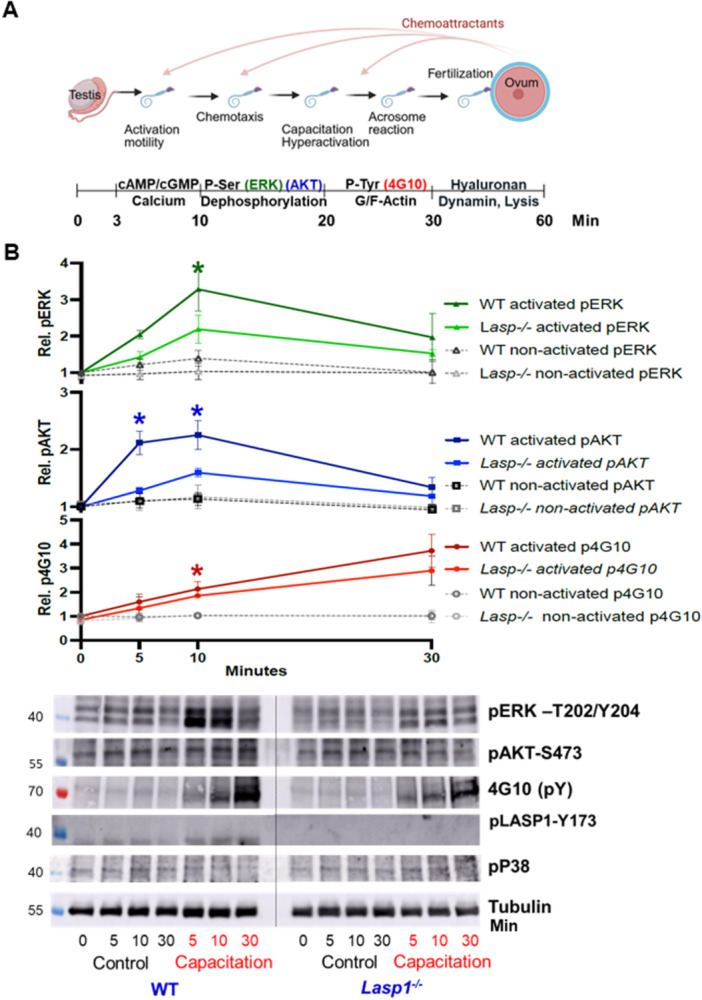
Protein phosphorylation during sperm capacitation in WT and LASP1‐deficient spermatozoa. (A) Model illustrating the temporal sequence of sperm activation with respect to phosphorylation and G/F‐actin polymerization (Created in BioRender. Butt (2026) https://BioRender.com/bbxe67). (B) Time‐dependent Western blot analysis of phosphotyrosine (4G10 antibody), phospho‐MAP‐kinases (pERK, pP38) and pAKT in wild‐type (WT) and *Lasp1* ^*‐/‐*^ mouse sperm without (control) and after activation (capacitation) with calcium/bicarbonate/albumin. Tubulin served as loading control. Statistical differences were examined by two‐way ANOVA test. Data are presented as mean ± SEM (*n* = 4). Significance *p* < 0.05 between groups at time point is indicated by an asterisk (_*_).

Capacitation is characterized by changes in protein kinase activity. Initiation of cAMP‐PKA is followed by downstream activation of MAPK cascade (including P38, ERK1/2 and AKT) and IP_3_ signaling (Kumar et al. 2024) (Figure 5A). LASP1 is known to bind AKT1 and enhances its activity (Butt et al. 2020; Subramaniyan et al. 2020). Therefore, we further investigated protein phosphorylation in spermatozoa using α‐tubulin as internal loading control as it remained constant during acrosome reaction (Feng et al. 2021).

Previous studies demonstrated ERK1/2‐T202/Y204 phosphorylation during capacitation, while P38 was not activated, suggesting a reciprocal regulation of sperm motility by these kinases (Almog et al. 2008). Our Western blot data confirmed these observations, and additionally showed that general protein tyrosine phosphorylation (detected by the anti‐phosphotyrosine antibody 4G10) and particular ERK1/2 phosphorylation were significantly reduced in LASP1‐knockout spermatozoa (Figure 5B). While ERK phosphorylation is seen within the first 5‐10 min of sperm capacitation, AKT phosphorylation is connected to hyperactivation along the entire tail (Sagare‐Patil et al. 2013) and started later (10–30 min) (Figure 5B). Again, phosphorylation was reduced in Lasp1‐depleted spermatozoa. Furthermore, we detected a weak Lasp1‐Y173 phosphorylation in the sperm of wild‐type mice. However, we were not able to detect PKA‐dependent phosphorylation, most likely due to low‐affinity binding capacity of LASP1‐T156 in whole cell lysate.

### 2.7 LASP1‐Knockout Affects Female Mice Fertility

No difference in the estrous cycle was observed between wild‐type and *Lasp1* ^‐/‐^ female mice, indicating normal mating behavior (*n* = 12 animals each).

**Figure 6:**
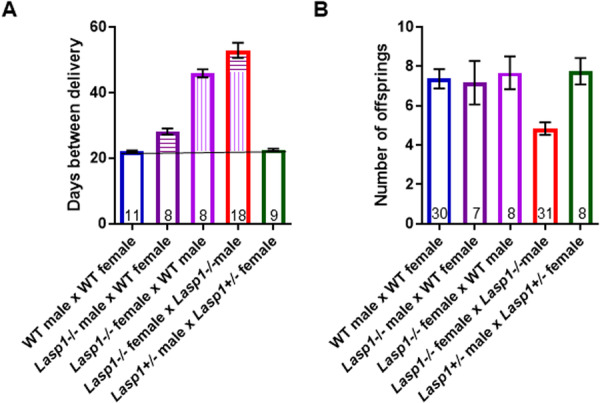
Male and female mice contribute to reduced fertility in *Lasp* ^*‐/‐*^ mice. Crossbreed mating studies were conducted by keeping the relevant pairs together for up to 30 weeks and recording (A) the days between litters and (B) the individual numbers of offsprings. Statistical differences for litters were measured by Kruskal‐Wallis test with *p* < 0.001 for all multiple comparisons. Statistical differences for offsprings were examined by unpaired Students t‐test. Data are presented as mean ± SEM. Number of breedings are shown in the bar chart.

Given the effects seen on male sperm in mice deficient in LASP1, we also investigated how the loss of LASP1 in female mice would perturb overall fertility. To explore this, we housed WT male with *Lasp1* ^*‐/‐*^ female mice, *Lasp1* ^*‐/‐*^ male mice with WT female mice, as well as heterozygous male and female mice for up to 30 weeks, and recorded days between two litters (Figure 6A) and number of pups born in each litter (Figure 6B). Heterozygous *Lasp1* ^+/−^ mice appeared normal with litter times of 22 ± 0.4 days (*n* = 9), and 7.7 ± 0.7 pups (*n* = 8). When *Lasp1* ^*‐/‐*^ male mice were intercrossed with WT female mice, females produced litters after 28 ± 0.9 days (*n* = 8). *Lasp1* ^*‐/‐*^ females mated with WT male mice showed significantly delayed litter times of 45 ± 1.1 days (*n* = 8). Surprisingly, litter size was normal in both crossbreedings: 7.3 ± 0.9 (*n* = 7) and 7.6 ± 0.8 (*n* = 8), respectively (Figure 6B).

In summary, the crossbreeding data indicate an additive effect of the *Lasp1* knockout on the reduced strain fertility. In addition to the spermatozoa phenotype in male mice, female mice are independently affected by *Lasp1* knockout; however, the underlying mechanism in female mice remains to be elucidated.

## 3 Discussion

In this study, we report for the first time that LASP1 is expressed in human and mouse spermatozoa and plays a role in sperm capacitation and the acrosome reaction.

LASP1 is co‐localized with actin in all four main parts of the sperm: head, neck, midpiece, and flagellum, but its major function is in the head, while tail motility is not affected. During capacitation, globular actin (G‐actin) polymerizes into filamentous actin (F‐actin). Prior to the acrosome reaction, proteins like gelsolin break down F‐actin to allow calcium‐triggered acrosomal exocytosis and egg penetration (Breitbart and Finkelstein 2018). PKA activation and tyrosine phosphorylation are necessary early processes during capacitation and actin polymerization (Breitbart et al. 2005). In this respect, LASP1 phosphorylation by PKA at S146 allows binding to F‐actin, but not G‐actin (Butt et al. 2003), and favors restructuring of the sperm head by stabilizing actin bundles (Nakagawa et al. 2006). Subsequent LASP1‐Y171 phosphorylation abrogates F‐actin stabilization and facilitates depolymerization (Lin et al. 2004). Loss of LASP1 significantly impairs the acrosome reaction, suggesting that the protein is important for sperm fertility. Furthermore, incomplete fusion of the acrosome membrane and the inner plasma membrane during the acrosome reaction hinders the surface expression of hyaluronan receptor CD44, as observed in LASP1‐deficient spermatozoa. Of note, male Drosophila melanogaster *Lasp1* null mutants are sterile (Lee et al. 2008). The flies show defects in the actin cytoskeleton attached to the spermatid plasma membrane.

Hyaluronic acid, the signaling molecule for CD44, is abundant in the cumulus oophorus and guides mature sperm to ensure fertilization ‐ acting as a checkpoint. Hyaluronic acid binding tests (HBA) exploit this process and select for mature sperm with DNA integrity for intracytoplasmic sperm injection (ISCI) to improve pregnancy (Huszar et al. 2003; Scaruffi et al. 2022). However, a direct causal relationship between reduced CD44 expression and male infertility remains uncertain.

Defects in the remodeling of the actin cytoskeleton during mammalian sperm capacitation and acrosome reaction are accompanied by multiple phosphorylation and dephosphorylation steps (Kumar et al. 2024). While P38 phosphorylation is not induced by progesterone stimulation (Almog et al. 2008; Sun et al. 2021), a Ras‐Raf‐specific ERK cascade is observed via G‐protein coupled receptors, PKC signaling, in conjugation with the bicarbonate, cAMP, Ca^2+^ pathway (Ickowicz et al. 2012). We confirmed the phosphorylation data: no P38 phosphorylation but increased early (5‐10 min) ERK1/2‐T202/Y204 phosphorylation and pAKT‐S473 activation, followed by delayed (30 min) Y‐phosphorylation (4G10 antibody) (Figure 5B). A recent study observed a time‐dependent PI3K/AKT‐T308 activation without S473 phosphorylation (Lee et al. 2025), however, we could not emphasize these data and exhibited increased AKT‐S473 phosphorylation during acrosome reaction.

In addition to the obvious and substantial effect of LASP1 on actin remodeling during acrosome reaction, the protein can directly bind to AKT1 and enhance S473 phosphorylation (Butt et al. 2020). In various malignancies, high LASP1 expression augments AKT1‐S473 phosphorylation and leads to tumor growth, cell proliferation, invasion and metastasis (Subramaniyan et al. 2020; Zhao et al. 2015). Physiologically, LASP1‐AKT1 interaction regulates NPC1L1 translocation in enterocytes during intestinal cholesterol uptake (Butt et al. 2024).

In summary, early motility and chemotaxis and the underlying Ca^2+^ balance and flagellar movement (Yoshida and Yoshida 2011) are not affected by LASP1, however, acrosomal phosphorylation signaling and cytoskeletal rearrangement to promote sperm maturation depend on this exceptional protein (Butt et al. 2022).

PKA is widely recognized as the dominant kinase regulating key processes in sperm activation and acrosome reaction while PKG has emerged as an additional cyclic nucleotide dependent regulator contributing to sperm signaling (Ickowicz et al. 2012). PKG is activated by cGMP, which is produced downstream of NO/soluble guanylyl cyclase (GC), or alternatively, by natriuretic peptides and particulate GC (Kuhn 2016). Human sperm express natriuretic peptide receptors, especially around the acrosomal region and the proximal flagellum and human PKG is discussed to be present in head and acrosomal region and to play an additional role in capacitation (Cisneros‐Mejorado et al. 2014). Since the N‐terminal leucine zipper differs significantly between PKGIα and Iβ, this could also explain differences in localization and anchoring.

Our crossbreeding data from *Lasp1* ^‐/‐^ mice indicate a significant contribution of female mice to the low birth rate and the long inter‐litter intervals. Successful blastocyst implantation can only occur when the endometrium is receptive during a short window with each menstrual cycle.

During early pregnancy in rats, LASP1 levels in the uterine luminal epithelial cells (UECs) increase during implantation (Nicholson et al. 2018). At this stage, LASP1 localizes apically and laterally within UECs and is associated with palladin, a known binding partner of LASP1 that is crucial for actin filament stabilization and plasticity (Rachlin and Otey 2006) suggesting a functional role in the morphological changes required for blastocyst attachment and implantation. In human UECs, talin‐1, vitronectin, and LASP1 build a complex and enforce endometrial adhesive capacity (Li et al. 2020).

LASP1 is also detected in extravillus trophoplasts (https://www.proteinatlas.org) ‐ cells that invade the uterine lining during early pregnancy to establish the blood supply to the placenta. A study utilizing EVT cell lines indeed demonstrated LASP1 expression in trophoblasts as well as its regulation by miRNA‐218, which directly targets the 3‘‐UTR of LASP1‐mRNA (Yu et al. 2021). In preeclampsia and under hypoxic conditions, miR‐218 is upregulated and suppresses the mRNA and protein levels of LASP1, thereby reducing trophoblast invasion and migration—processes crucial for placental development (Fang et al. 2017). However, further investigations are required to more precisely elucidate the functional significance of LASP1 in female animals during early pregnancy—or possibly during uterine regeneration—a process that is highly dependent on F‐actin and may contribute to long inter‐litter intervals.

In summary, this study provides new insight into the functional importance of LASP1 in reproductive biology. The present work mainly focused on LASP1 and its functional role in spermatozoa. The protein is expressed in all parts of the spermatozoa and co‐localizes with F‐actin. During capacitation and acrosome reaction, LASP1 stabilizes the actin bundles in the sperm head and builds up the equatorial ring segment, a specialized region the plasma membrane in the mature sperm and important for egg fusion.

## 4 Materials and Methods

### 4.1 Mouse Breeding

Lasp1−/− mice (strain #008789) and Bl6 control mice were purchased from the Jackson Laboratory. The mice were kept together from week 8 to week 50 without delaying mating. For statistical analysis, only the days between the 2nd and 3rd litters and between the 3rd and 4th litters were considered.

### 4.2 Mouse Sperm Preparation

All experiments were performed in 12–16 weeks old male mice. Mice were anesthetized using isofluorane and euthanized. Studies are in agreement with the Directive 2010/63/EU of the European Parliament and in accordance with the guidelines of the University Clinic of Wuerzburg and the local authorities (Regierung von Unterfranken, Würzburg, Germany).

For sperm isolation, caudae epididymidis were dissected and transferred into 500 µl gassed (5% CO_2_) sperm pre‐incubation medium (30 mM Hepes pH 7.4, 137 mM NaCl, 5 mM KCl, 1 mM MgCl_2_, 1.2 mM KH_2_PO_4_, 10 mM Glucose, 10 mM Na‐lactate, and 1 mM Na‐pyruvate) in 24 well plates. The caudae epididymides were cut five to six times and plate was placed at 37°C/5% CO_2_ in an incubator for 15 min equilibrating. Sperm cells were collected and counted.

Sperm activation was induced by adding 1 mg/ml BSA, 2 mM CaCl_2_, and 25 mM NaHCO_3_.

### 4.3 Mouse IVF

IVF was performed according to the validated protocols of the Mary Lyon Center at MRC Harwell based on the work published by Takeo et al. 2011.

Briefly, dishes were overlaid with mineral oil and pre‐incubated. Fresh sperm cells were collected by dissecting caudae epididymidis and transferring it into a 90 µl drop of pre‐incubation medium (TYH + MBCD). Oocytes were harvested by dissecting the oviducts from superovulated female mice, releasing the cumulus masses into the oil and dragged into the fertilization drop. After at least 30 min of exposure to the fertilization drop (HTF + GSH) and equilibration of the spermatozoa (60 min) the sperm suspension was added (3–5 µl). After 10 min, sperm motility, concentration and compactness of corona cells within the cumulus masses were judged. To allow fertilization, all dishes were incubated for 4 h, before collecting and washing the oocytes. After overnight culture, 2‐cell embryos were separated from unfertilized or degenerated oocytes and transferred into culture medium (KSOM) before cryopreservation or harvested into Laemmli buffer for Western blot analysis.

The extended protocols are provided for download by INFRAFRONTIER (https://www.infrafrontier.eu/emma/cryopreservation-protocols/).

### 4.4 Human Sperm Preparation

Sperm samples were collected by masturbation after a period of sexual abstinence of 2–5 days. After complete liquefaction at room temperature (RT), semen analysis was performed according to World Health Organization criteria. Briefly, 10 µl of well‐mixed semen was transferred to a Makler chamber (CooperSurgical, CT, USA) for analysis. Seminal parameters were evaluated (Microscope CX43, Olympus, Japan). For density gradient separation, an 80% one‐layer gradient protocol was used (Isolate and MHM, FUJIFILM Irvine Scientific, CA, USA). 1.0–1.5 mL of the liquified semen was gently dispensed into the tube onto the 1 ml 80% Isolate layer in at least four 15 mL conical centrifuge tubes. After centrifugation for 20 min at 300*g* (Rotofix 32 A, Hettich, Germany) the layers were removed just below the surface of the liquid. Sperm pellet was transferred to a new tube and washed for two times with 3 mL of Sperm Washing Medium (MHM, FUJIFILM Irvine Scientific, Santa Ana, CA, USA) and centrifuged 200*g* for 10 min. Supernatant was removed and sperm pellet resuspended with MHM and adjusted to the desired concentration. For activation, 1 mg/ml human albumin was added.

### 4.5 Analysis of Sperm Morphology and Motility

Spermatozoa were analyzed for head, midpiece, and tail defects using a light microscope (Zeiss Axiovert, Jena, Germany). In addition, full length and sperm head area were measured. More than 50 sperm were observed for each animal (*n* = 10).

Semen analysis was performed using an automated sperm analyzer (CASA; Hamilton Thorne, IVOS II, Beverly, MA, USA). Up to 3 µl sperm sample was pipetted into a Leja counting chamber (Nieuw‐Vennep, The Netherlands) to determine total sperm concentration, percentage of overall motile sperm and percentage of progressively swimming sperm.

### 4.6 Sperm Chemotaxis Assay

Chemotaxis was measured according to the protocol by Burnett et al. (2011). Sperm from 10 mice were used in the experiments. Acetylcholine was used as a chemoattractant. The µ‐slide (ibidi, #80166, Gräfelfing, Germany) was loaded with 100 µl sperm medium and maintained at 37°C. 10 µl of 50 mM acetylcholine (Sigma‐Aldrich, A6625) was added into the outlet, covered with 1 µl mineral oil and kept for 5 min to form a concentration gradient. After forming the gradient, 10 μl of sperm suspension (1 x 105) was loaded into the inlet and covered with 1 μl of mineral oil. The slide was left for 10 min. Total distance reached by at least 10 spermatozoa was measured using an optical microscope at 10x magnification.

### 4.7 Immunoprecipitation

LASP1 immunoprecipitation (IP) in mouse sperm was performed by rotation of 1 x 10^7^ cells in 500 µl RIPA^+^‐buffer (20 mM Tris pH 7.4, 150 mM NaCl, 1% sodium‐deoxycholate, 1% Triton‐X‐100, 0.1% SDS, 1 mM sodium orthovanadate, 10 mM sodium‐pyrophosphate, 10 mM EDTA and protease inhibitor mixture) with 2 µg in‐house LASP1 antibody #1343 for 2 h at 4°C followed by incubation with A/G sepharose beads (Santa Cruz #2003) for another 2 h at 4°C. After washing the sepharose beads two times with ice‐cold PBS, F‐actin binding to LASP1 was analyzed by Western blot.

### 4.8 Western Blot Analysis

Sperm (2 million/lane) and oocytes (20/lane) were subjected to 10% SDS‐PAGE, blotted onto nitrocellulose (GE Healthcare, Freiburg, Germany) and analyzed by immunoblotting with the following antibodies: In‐house total LASP1 (#1343 and #pan1418) (Butt et al. 2003), pLASP1‐Y171 (#1180) (Frietsch et al. 2014) (corresponding to mouse pLASP1‐Y173 with identical epitope amino acid sequence) and pLASP1‐T156 (#1500) (Supplorting Information Figure S3); α‐Tubulin (Sigma‐ T9026); Actin (Sigma‐Aldrich, A2066); PKA C‐subunit (Cell signaling #4782, Danvers, MA); in‐house PKG1α and 1β; pP38 (Cell Signaling, #9211); pAKT1 (Cell Signaling, #4060); 4G10 (Cell Signaling, #96215); CD44 (Proteintech, #15675); pERK (Cell Signaling, #9101).

Before overnight incubation with primary antibodies, membranes were blocked with 5% dry milk or BSA (Biorad, Munich, Germany) in TBS with 0.1%Tween for 1 h at RT. Immunoblots were probed with the corresponding secondary horseradish peroxidase conjugated antibody purchased from Biorad and developed using enhanced chemiluminescence reagent (GE Healthcare, Freiburg, Germany). Images were taken using the Amersham Imager 600 (GE Healthcare) and quantified by the Image QuantTL software (GE Healthcare).

### 4.9 Sperm Activation and Immunocytochemistry

Human and mouse spermatozoa were isolated as described above, and capacitation was started by adding 1 mg/ml human albumin, respectively 1 mg/ml BSA, 2 mM CaCl_2_, and 25 mM NaHCO_3_. For the time points indicated, 5 µl sperm was smeared on poly‐l‐lysine coated coverslips (Epredia, Breda, The Netherlands), dried for 3–5 min at RT, fixed for 15 min with ice‐cold 95% ethanol on ice, washed with H2O and stored at RT.

For PNA staining, slides were washed 4x with PBS and incubated 30 min in the dark with Alexa‐488 conjugated PNA (Lectin‐PNA‐488 #L21409, Molecular Probes, Willow Creek, OR; 1:100).

For LASP1 and actin staining, non‐specific binding was blocked with DAKO blocking buffer (1 h at RT) prior to overnight incubation with primary LASP1 antibody (in house #pan1418, directed against the dephospho‐Y171 site. 1:50) at 4°C. After three PBST (0.05% Tween‐20) washes, sections were incubated with Alexa Fluor 555 (Thermo Fisher, Karlsruhe, Germany; 1:500) conjugated secondary antibody for LASP1 staining and Oregon‐green Phalloidin (InvitrogenTM, Thermo Fischer; 1:30) for actin staining (2 h at RT in the dark).

After staining, slides were washed 4x with PBS and coverslipped using DAPI‐containing Vectashield mounting medium (Vector Laboratories, Burlingame, USA).

Fluorescent images were acquired using the Thunder Imaging system (Leica, Wetzlar, Germany).

### 4.10 Flow Cytometry of Hyaluronan Receptor (CD44)

Immediately after isolation, and 30 min and 60 min after activation, spermatozoa were fixed with 1% PFA for 15 min on ice and washed with PBS. When all samples were available, cells were stained with anti‐CD44 PE (clone: IM7, eBioscience, Thermo Fisher, 1:100 in PBS). For total CD44 expression, cells were permeabilized using BD Cytofix/Cytoperm kit (BD Bioscience, Heidelberg, Germany) and subsequently stained with anti‐CD44 PE. Cells were recorded on a FACS Celeste 5V‐4B‐3R (BD Bioscience) equipped with a high‐throughput sampler (BD Bioscience) and analyzed in FlowJo 10.10.0 (BD Bioscience). Mean fluorescence intensities (MFI) of PE were calculated gated on spermatocytes according to FSC‐SSC.

### 4.11 Determination of Estrous Cycle

The estrous cycle was studied using cells removed by gentle mechanical disruption following vaginal washes, stained with hematoxylin/eosin solution and analyzed by light microscopy according to (McLean et al. 2012). Samples were classified as follows: proestrus, nucleated epithelial cells with occasional leukocytes and small degenerative nuclei cells; estrus, numerous cells from squamous epithelium and small epithelial cells; metestrus, numerous leukocytes and small cornified epithelial cells, and diestrus, mucus and nucleated cells.

### 4.12 Statistical Analysis

Data represent mean ± SEM. Comparisons were performed via unpaired Student's *t*‐test for normally distributed data, Mann‐Whitney test for non‐normally distributed data (D'Agostino test), using Prism software (GraphPad, Version 10). Differences were considered statistically significant at *p* < 0.05.

## Author Contributions

Alicia Bender did sperm statistics and chemotaxis measurements, Kilian Andress performed immunocytochemistry on human and mouse spermatozoa; Michael R. Boesl performed IVF experiments and motility assays; Burkhard Kneitz, Claudia Staib and Adriane Woehl‐Wenigerkind collected and prepared human sperm; Hendrik Bartolomaeus measured CD44 expression; Alma Zernecke interpreted results and was involved in drafting the manuscript, Elke Butt conceived and designed research, performed all Western blots, and drafted the article. All authors edited and revised the article.

## Ethics Statement

Studies are in agreement with the Directive 2010/63/EU of the European Parliament and in accordance with the guidelines of the University Clinic of Wuerzburg and the local authorities (Regierung von Unterfranken, Würzburg, Germany).

## Conflicts of Interest

The authors declare that they have no conflict of interests.

## Supporting information

Supporting File 1

## Data Availability

The data that support the findings of this study are available from the corresponding author upon reasonable request.
