# Supplementary material for: LASP1, a Novel Protein in Spermatozoa and Acrosome Reaction
Source: Mol Reprod Dev. 2026 Jul 6;93(7):e70127. doi: 10.1002/mrd.70127 (PMC13334231; doi:10.1002/mrd.70127)
Supplement: Supplementary file 1 — Supporting File 1 [file MRD-93-e70127-s001.pptx]

## Slide 1
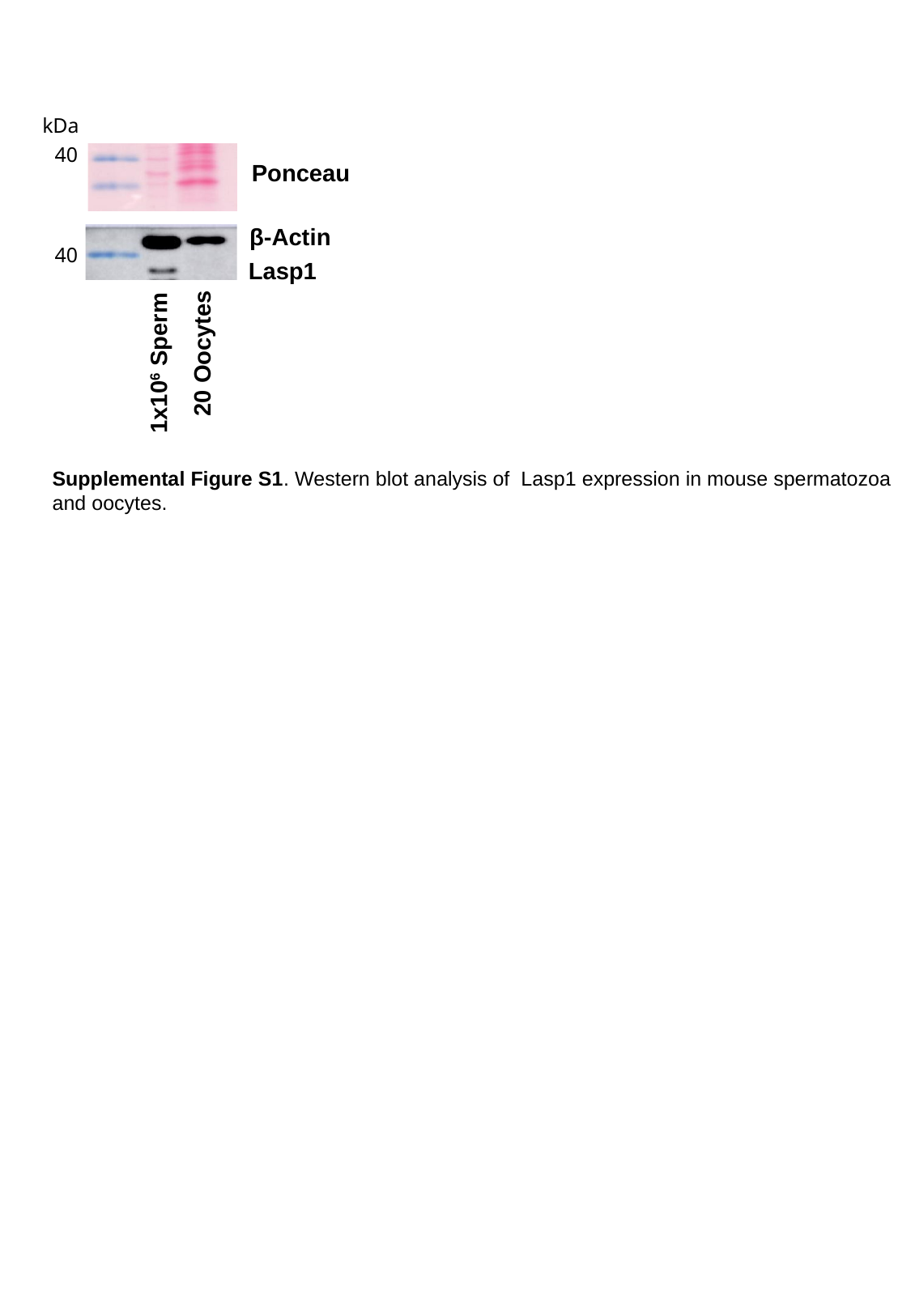

kDa
40
Ponceau
β-Actin
40
Lasp1
20 Oocytes
1x10^6^ Sperm
Supplemental Figure S1. Western blot analysis of Lasp1 expression in mouse spermatozoa and oocytes.

## Slide 2
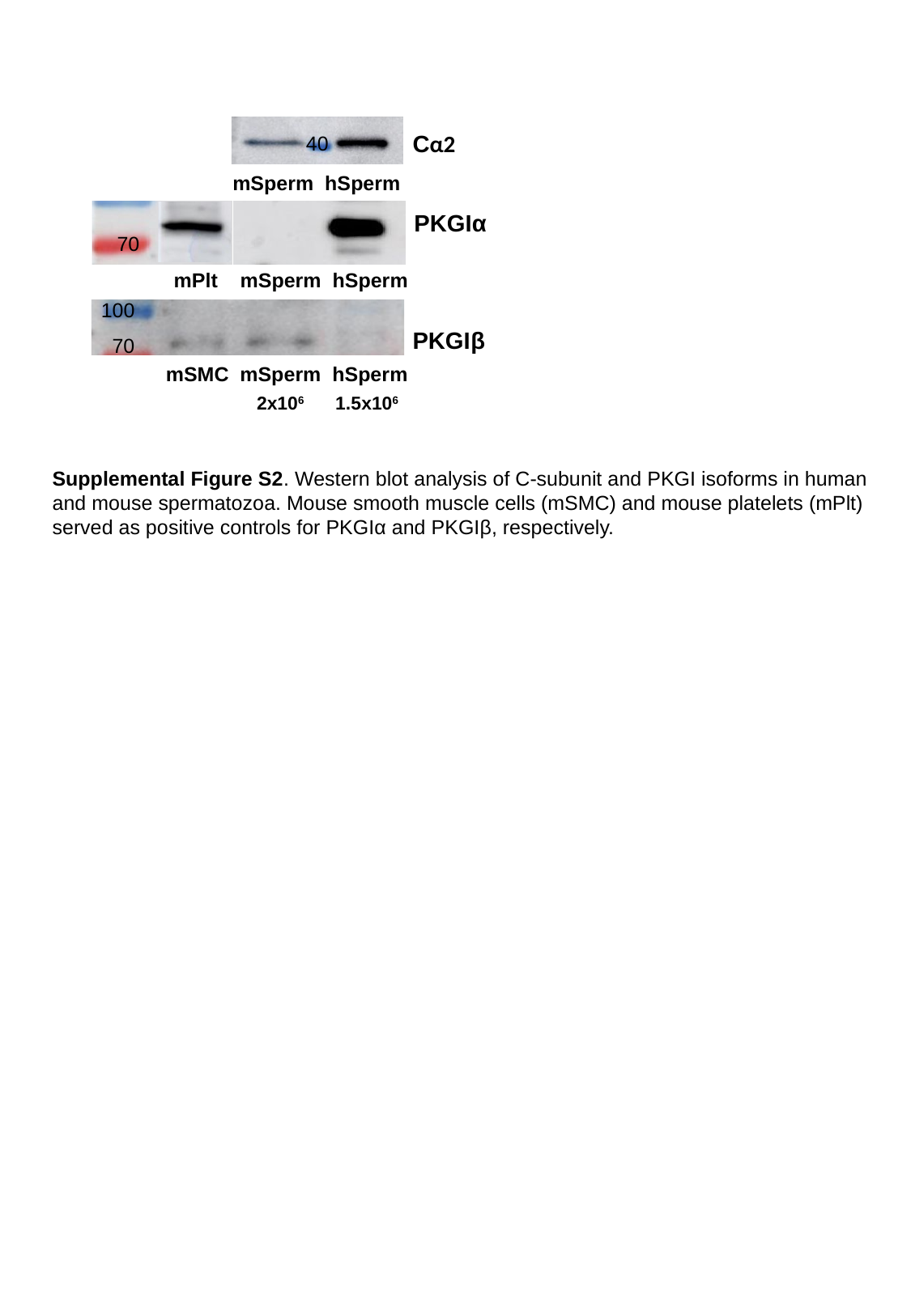

Cα2
40
mSperm hSperm
PKGIα
70
 mPlt mSperm hSperm
100
PKGIβ
70
 mSMC mSperm hSperm
 2x10^6^ 1.5x10^6^
Supplemental Figure S2. Western blot analysis of C-subunit and PKGI isoforms in human and mouse spermatozoa. Mouse smooth muscle cells (mSMC) and mouse platelets (mPlt) served as positive controls for PKGIα and PKGIβ, respectively.

## Slide 3
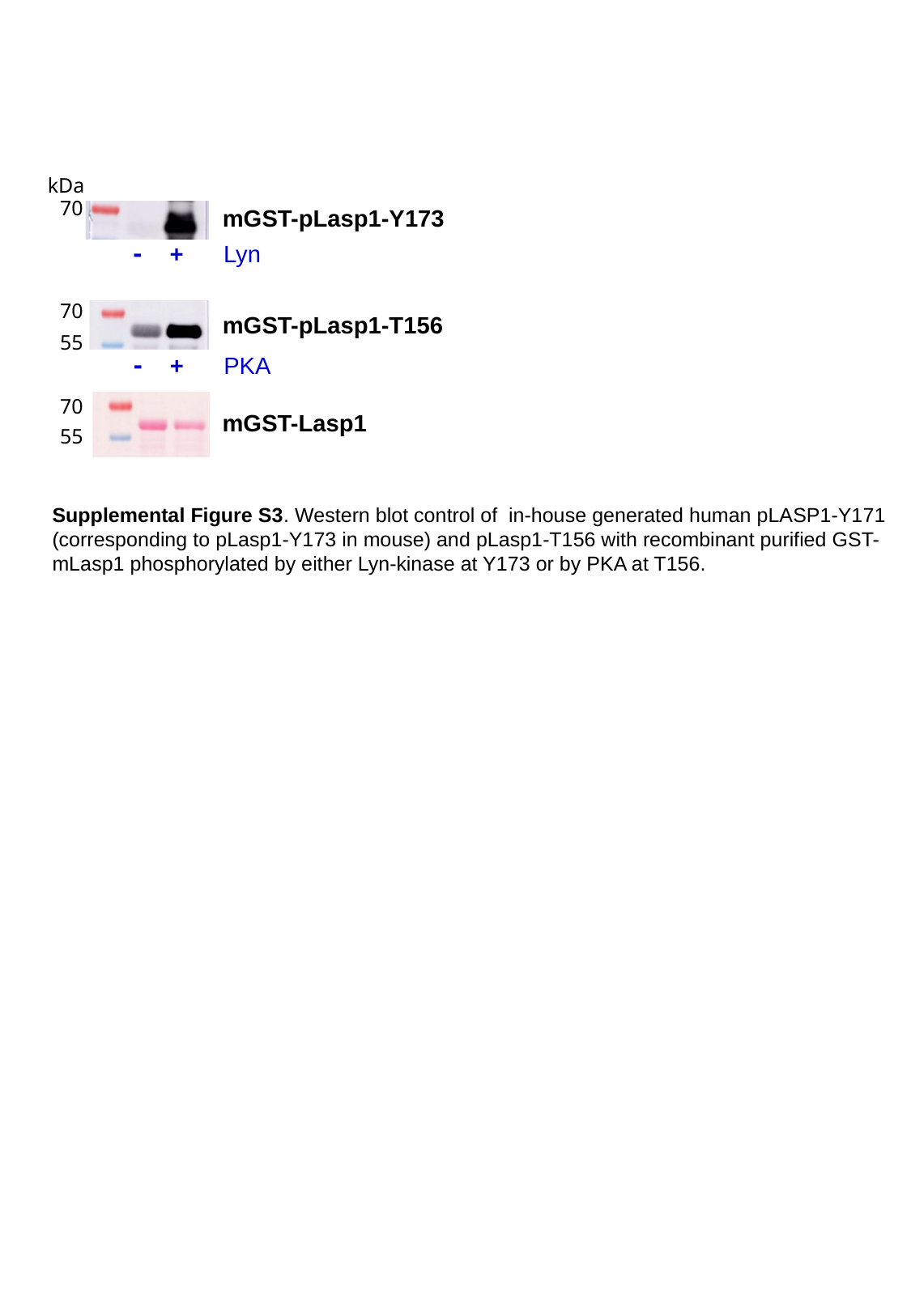

kDa
70
mGST-pLasp1-Y173
- + Lyn
70
mGST-pLasp1-T156
55
- + PKA
70
mGST-Lasp1
55
Supplemental Figure S3. Western blot control of in-house generated human pLASP1-Y171 (corresponding to pLasp1-Y173 in mouse) and pLasp1-T156 with recombinant purified GST-mLasp1 phosphorylated by either Lyn-kinase at Y173 or by PKA at T156.
